# Nanosecond time-resolved characterization of a pentacene-based room-temperature MASER

**DOI:** 10.1038/srep41836

**Published:** 2017-02-07

**Authors:** Enrico Salvadori, Jonathan D. Breeze, Ke-Jie Tan, Juna Sathian, Benjamin Richards, Mei Wai Fung, Gary Wolfowicz, Mark Oxborrow, Neil McN. Alford, Christopher W. M. Kay

**Affiliations:** 1 London Centre for Nanotechnology, University College London, 17-19 Gordon Street, London WC1H 0AH, UK; 2 Institute of Structural and Molecular Biology, University College London, Gower Street, London WC1E 6BT, UK; 3 School of Biological and Chemical Sciences, Queen Mary University of London, Mile End Road, London E1 4NS, UK; 4 Department of Materials, Imperial College London, London SW7 2AZ, UK

## Abstract

The performance of a room temperature, zero-field MASER operating at 1.45 GHz has been examined. Nanosecond laser pulses, which are essentially instantaneous on the timescale of the spin dynamics, allow the visible-to-microwave conversion efficiency and temporal response of the MASER to be measured as a function of excitation energy. It is observed that the timing and amplitude of the MASER output pulse are correlated with the laser excitation energy: at higher laser energy, the microwave pulses have larger amplitude and appear after shorter delay than those recorded at lower laser energy. Seeding experiments demonstrate that the output variation may be stabilized by an external source and establish the minimum seeding power required. The dynamics of the MASER emission may be modeled by a pair of first order, non-linear differential equations, derived from the Lotka-Volterra model (Predator-Prey), where by the microwave mode of the resonator is the predator and the spin polarization in the triplet state of pentacene is the prey. Simulations allowed the Einstein coefficient of stimulated emission, the spin-lattice relaxation and the number of triplets contributing to the MASER emission to be estimated. These are essential parameters for the rational improvement of a MASER based on a spin-polarized triplet molecule.

Recently a prototype of a pulsed MASER operating at room temperature and with no static applied magnetic field was demonstrated^1^,^2^. The system is based on a single crystal of pentacene-doped para-terphenyl (pentacene: *p*-terphenyl) as gain medium loaded in a single mode microwave resonator. The emission frequency (1.45 GHz) is close to that of the hydrogen MASER (1.42 GHz), making it an alternative for practical applications. In order to expand the scope of room temperature MASERs and allow a larger choice of emission frequencies, other acenes have been considered theoretically as substitutes of pentacene^3^ while inorganic materials with high spin ground states, such as NV centres in diamond (S = 1)^4^ and defects in SiC (S = 3/2)^5^, have been proposed as candidates for room-temperature microwave quantum emitters due to their long lifetime and spin polarisation. However, none of these suggestions have yet been experimentally demonstrated in working devices, and therefore, to date, the sole working room-temperature solid-state MASER is based on pentacene: *p*-terphenyl.

The photo-physics of pentacene: *p*-terphenyl and the key concepts that lie at the heart of its utility as a gain medium are depicted in Fig. 1. The promotion of pentacene to its first excited singlet state (S_1_) by absorption of an appropriate photon (absorption maximum, λ_max_, at 590 nm) results in the second triplet state (T_2_) being populated following inter-system crossing (ISC). Since S_1_ and T_2_ are almost resonant, the quantum yield of triplet is high, approximately 0.625^6^. Moreover, the rates of ISC to the three triplet sub-levels are anisotropic leading to non-Boltzmann populations of the triplet sub-levels^7^,^8^. T_2_ has a short lifetime and relaxes rapidly to the first excited triplet, T_1_, with conservation of the relative spin-populations.

T_1_ has several key properties that make it a suitable gain medium for MASER operation: (a) the dipolar coupling between the two unpaired electrons, described by the *Zero field splitting* (*ZFS*) terms *D* and *E*, splits the energy of the sub-levels even in zero external magnetic field giving an energy gap ≈1.45 GHz between uppermost (X) and lowest (Z) sub-levels^7^,^8^; (b) the anisotropy of the ISC results in an initial population ratio between the uppermost (X) and the lowest (Z) levels of 9.5:1, and hence a considerable population inversion; (c) the triplet sub-levels have substantial lifetimes of X ~ 22 μs, Y ~ 44 μs and Z ~ 1000 μs at room temperature^9^; (d) the spin-lattice relaxation between the three triplet sub-levels (>330 μs at room temperature^9^) is slow enough so it does not compete with stimulated emission.

Thermal photons inside the gain material (crystal and resonator) act as seeds for stimulated emission. The use of a resonator with a single mode ensures that only photons with the same frequency (within the bandwidth) and phase are amplified, thus providing monochromaticity, coherence and polarization. Consequently, if the gain (through stimulated emission) is larger than the losses (through re-absorption of photons or leakage via the output coupler) then an exponential build up of microwave photons in the resonator can occur. Although the published studies used pulsed optical excitation, the length of the pulses, ~350 μs^1^ and ~20 μs^2^ respectively, were comparable to the spin evolution of the pentacene triplet, which complicates analysis and interpretation of the dynamics of the MASER emission.

Here, in order to monitor the microwave output of the MASER without the complication of quasi-continuous illumination, we employed short (5 ns) and high-energy (0–8 mJ/pulse) pulses in conjunction with a strontium titanate (STO) resonator that has recently been described^2^. This approach allowed us to observe how the build up, intensity, and decay of the microwave output depend on the optical excitation energy. The analysis of our experiments using a Lotka-Volterra (L-V), also known as predator-prey, model, in which the spin-polarization is the prey and the resonator mode is the predator, provides fundamental insights into the underlying physics. We also show that the MASER output can be conveniently stabilized through seeding with an external frequency source.

## Results

### MASER threshold measurement

MASER emission following pulsed laser excitation was directly detected by an oscilloscope (6 GHz bandwidth) without signal averaging. A typical output following a single laser pulse with 3.3 mJ excitation energy is shown in Fig. 2a. A fast-Fourier transform based spectral estimate was used to compute the frequency and power components of the time-domain signal, and indicates that as expected, the maximum signal appears at ~1.45 GHz (Fig. 2b) directly confirming that the MASER emission originates from the X ↔ Z transition within the triplet sublevels^7^,^8^. Taking a slice through the contour plot at 1.45 GHz allows the temporal response to be extracted (Fig. 2c). As the laser energy and hence the output varies from pulse to pulse, one hundred consecutive time traces (2 Hz repetition rate) were recorded at each nominal laser energy, see Supplementary Video 1 for a typical data set.

Figure 2d depicts the temporal response of the MASER as a function of laser energy over the range 0–8 mJ. The time traces show a microwave burst that reaches a maximum several microseconds after the laser flash and, although at each laser power there is a distribution of both amplitude and delay, a clear trend is observed: at higher laser power, the signal amplitude is larger and the delay is shorter.

The variation in the MASER output is likely to be caused by a combination of fluctuations in the laser excitation energy and thermal heating of the pentacene: *p*-terphenyl crystal and the STO resonator, which changes the frequency of the resonator mode (STO has a large temperature coefficient of frequency of 1700 ppm K^−1^)^10^,^11^. At each average laser energy, there is a distribution of excitation energy, which appears to contribute to the distribution of the peak power and delay. To understand the nature of the distributions, histograms of the laser energy, peak power, delay and frequency at each mean laser energy were constructed and compared to Gaussian distributions, see Supplementary Fig. S1. One-sample Kolmogorov-Smirnov tests confirmed that none of the distributions deviate significantly from normality.

The peak power as a function of laser energy over the range 0–8 mJ/pulse is plotted in Fig. 2e (blue dots). The data indicate there is an optical excitation threshold (~3.2 mJ) below which no microwave output is detected, and above which the peak output power increases linearly. Linear regression gives an optical-to-microwave photon conversion efficiency of ~8 μW MASER output per mJ laser excitation (Fig. 2e, red line).

The dynamical behavior of the system may be accounted for by a pair of first order, non-linear, differential equations, derived from the L-V model, often referred to as a Predator-Prey mode^12^:


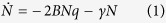



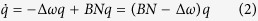


where: *N* is the spin-polarized population inversion (the prey), *B* is the Einstein coefficient of stimulated emission, *q* is the resonator mode photon population (the predators), *γ* is a phenomenological decay rate for the spin polarization and Δ*ω* the cavity mode linewidth, Δ*ω* = *ω*/*Q. ω* is the angular frequency and *Q* is the loaded quality factor. The coupling coefficient, *k* = 0.3, for the output port was implicitly included in Eqs (1) and (2) through the measured linewidth and the modelling calculated the MASER output power as a function of cavity photon number through an expression dependent on *k*.

From the Bose-Einstein occupation function for bosons 
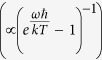
, and with *Q* = 4900, Δ*ω* = 1.86 MHz, the thermal photon population of the resonator at room temperature at time *t* = 0 is calculated to be *q*_0_ = 4282. So by varying the initial population inversion, *N*_0_, B and *γ*, predictions for the delay, amplitude and MASER emission profile may be obtained by solving the L-V equations. The best agreement between the predictions (Fig. 2d and f, red line) and the experimental values (Fig. 2d, solid black lines and Fig. 2f, blue dots) was obtained with an Einstein coefficient *B* = 11 ± 1 × 10^−8^ s^−1^, a spin-lattice relaxation rate *γ* = 7 ± 1 × 10^4^ s^−1^ while varying *N*_0_ over the range: 4.2–6.6 × 10^13^. Note that the data presented in Fig. 2d and f were simulated simultaneously in order to obtain a single set of parameters and increase the accuracy of the approach. Simulations for the upper and lower boundary of *B* and *γ* are included in Fig. 2f (dotted lines). As expected for decay functions, the upper boundary (slower decay) corresponds to the combination of smaller numerical values of *B* and *γ*.

The value for *B* derived from the numerical fitting is in excellent agreement with that predicted from time-dependent perturbation theory, 11.3 × 10^−8^ s^−1^ (see Materials & Methods). The spin lattice-relaxation time (*γ*) derived here in zero-field appears to be 5–10 times faster than the corresponding values (1–2 × 10^4^ s^−1^ depending on the transition) measured with electron paramagnetic resonance spectroscopy at 9 GHz^7^.

Simulated time traces with delay equal to the mean delay of each experimental data set (Fig. 2d, red lines) illustrate that the dynamics are reasonably described by the L-V equations, with the given parameters. Nonetheless, it is apparent that the model proposed, while describing satisfactorily the rising of the MASER emission, agrees more poorly with its decay. We expect this discrepancy stems from the single, phenomenological relaxation rate for the spin polarization.

Knowing N_0_, and given the anisotropy of the triplet populations P_x_:P_y_:P_z_ = 0.76:0.16:0.08 and the ISC efficiency of 0.625^6^, the maximum number of triplets, 3 × 10^15^, may be calculated. From the mass of the pentacene: *p*-terphenyl crystal (78.8 mg), and the ~0.01% mol/mol concentration of pentacene molecules it contains, the total number of pentacene present can be estimated to be 2 × 10^16^. However, as the crystal is longer (8 mm) than the diameter of the laser beam (4 mm) and hence only molecules in a central portion of the crystal are excited, the maximum number of triplets could be 10^16^, which is only slightly larger than the maximum number of observed triplets. We note that a previous study on light penetration in single crystals of pentacene: *p*-terphenyl suggested that only the outer layer is effectively excited by the laser pulse^6^. Together, with reflections at the surface of the crystal and the STO resonator, we conclude that these factors account for the smaller number of triplets observed and the maximum that could be formed if every pentacene molecule in the sample was excited.

### MASER seeding measurement

In laser systems, an established way to increase the output and stabilize it in terms of both the frequency and timing of the output pulse is to inject radiation of appropriate wavelength into the oscillator. As depicted in Fig. 3, a second antenna, weakly coupled (~−27 dB) to the resonator injected continuous microwave radiation at 1.45 GHz for a range of powers from −70 to −20 dBm and at constant laser excitation energy (6.7 mJ/pulse).

The expected effect is indeed observed: as the seeding power increases above −70 dBm at the source, the output energy increases concomitant with a decrease both in the absolute delay and the variability thereof. From the dependence of the peak power versus seeding power a linear increase in the output power of 0.44 μW dBm^−1^ can be calculated.

The L-V equations may again be invoked to simulate the dynamical behavior of the system under seeding conditions. The only variable in the system is *q*_0,_ which has a contribution from the seeding photons in addition to the thermal photons. Hence, by solving the L-V equations, with constant N_0_, *B* and *γ*, the predicted delay can be matched to the mean observed delay, and the number of seeding protons may be obtained for each nominal seeding power.

For example, at −60 dBm, the number of seeding photons required to give the observed delay is 2 × 10^6^. −60 dBm equates to 1 nW and therefore to 10^15^ photons s^−1^, at 1.45 GHz. Knowing the cavity linewidth, Δ*ω*, the photon flux with a coupling factor of unity would be 5 × 10^8^. However, the coupling was estimated to be −27 dB. Hence, the actual microwave photon flux should be 10^6^, which matches closely the measured value of 2 × 10^6^. Besides providing a straightforward method to stabilize the MASER output, these experiments also indicate that the fluctuation of the thermal photon population of the resonator, *q*_*0*_, is responsible, at least partially, for the observed variability in the MASER output.

## Discussion

We have examined the response of a pentacene-based room-temperature MASER to nanosecond duration optical pumping, directly detecting the microwave output with a high bandwidth digital oscilloscope. Noteworthy is a maximum MASER output of 50 μW. From MASER emission at different optical pumping powers, we were able to determine directly the threshold value, i.e. the value at which the gain of the microwave medium is exactly counterbalanced by the losses in the system. Moreover by means of the L-V model we could determine the Einstein coefficient of stimulated emission (*B*), the spin polarization relaxation rate (*γ*), and the number of triplets (*N*_*0*_). These parameters define the overall efficiency of the system and intimately depend on the active gain medium.

**Figure 1:**
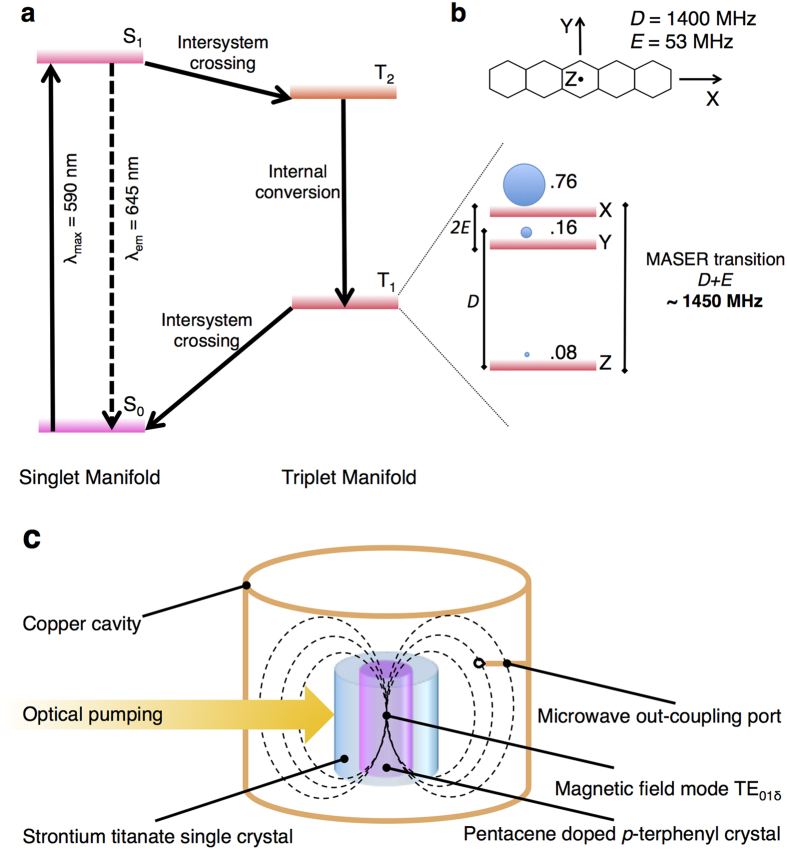
Basic principles and construction of a pentacene-based room temperature MASER. (**a**) Simplified Jablonsky diagram for pentacene depicting the generation of spin polarization following photo-excitation. The photo-physical processes involved are represented by arrows. (**b**) Molecular structure of pentacene overlaid with the orientation of the *ZFS* axes system and energy order of the triplet sublevels and relative spin populations; both the *ZFS* parameters, *D* and *E*, are assumed to be positive as previously reported^7^,^8^. (**c**) Schematic representation of the experimental apparatus, adapted from ref. 2.

**Figure 2:**
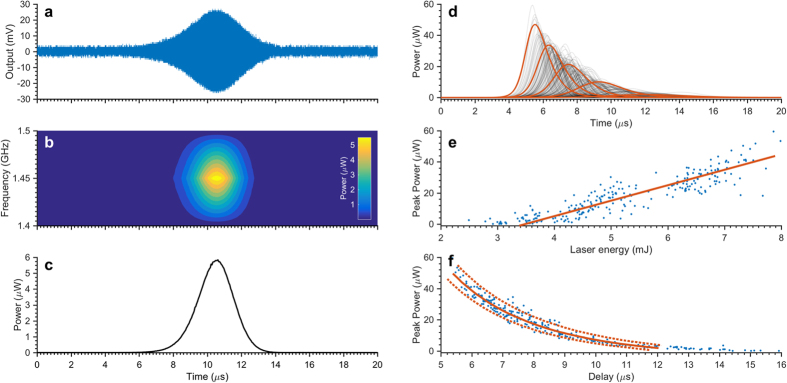
Dependence of the MASER emission on laser energy excitation energy. (**a**) Experimental single MASER emission recorded at 3.3 mJ excitation energy. (**b**) Frequency-power analysis of the MASER emission reported in panel a. (**c**) Output power of the MASER emission taken at the maximum of panel b. (**d**) Experimental temporal MASER emission as a function of laser pump energy (black lines) as a function of pumping laser energy. The red lines represent the best numerical fitting to the experimental data obtained according to the L-V model^12^. (**e**) MASER emission peak power as a function of laser pumping energy. Each blue dot represents the experimental peak power as extracted from the curves reported in panel d. The red line is the linear fitting to the data, the slope of the line relates to the optical-microwave photon conversion efficiency: ~8 μW mJ^−1^. (**f**) MASER emission peak power as a function of the time delay between the laser flash and the emission maximum (blue dots). The solid red line represents the best agreement between the experimental data and prediction according to the L-V model^12^ with an Einstein coefficient *B* = 11 × 10^−8^ s^−1^, a decay rate for the spin polarization *γ* = 7 × 10^4^ s^−1^ while varying *N*_0_ over the range: 4.2–6.6 × 10^13^. The upper and lower boundaries, corresponding to the combinations *B* = 10 × 10^−8^ s^−1^, *γ* = 6 × 10^4^ s^−1^ and *B* = 12 × 10^−8^ s^−1^, *γ* = 8 × 10^4^ s^−1^ respectively, are also depicted (dotted red lines).

**Figure 3:**
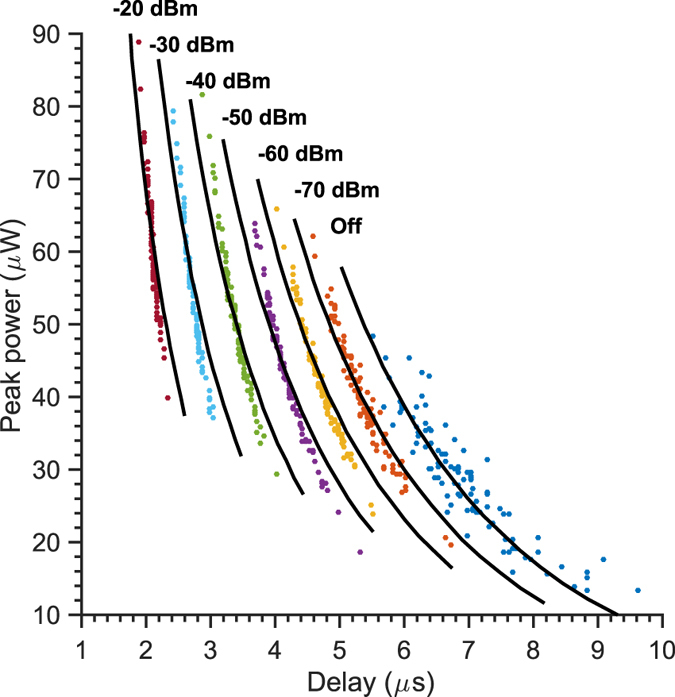
Dependence of the MASER time response as a function of seeding power. Variation of seeding power over the range −20 to −70 dBm. The response without seeding is also reported as a reference (blue dots). The black lines depict the L-V dynamic simulations under seeding conditions. Data recorded at constant laser excitation energy of 6.7 mJ/pulse.

Other physical-chemical parameters play a role in MASER performance and must be considered if alternative gain media to pentacene are sought. In particular:

(i) The ISC efficiency should be high so that less of the pump energy is wasted through fluorescence or internal conversion; the latter route is particularly important, as it leads to heating of the sample; (ii) the anisotropy of the ISC should ideally populate only the uppermost state, thus leading to total inversion; (iii) the triplet recombination rates should be long enough so that masing can occur, but not too long, to minimize the lag time between consecutive cycles; (iv) the spin-lattice relaxation rate should be slow, otherwise the populations will relax to Boltzmann equilibrium and the inversion will be lost. These properties, together with the *ZFS* parameters (Fig. 1) of the triplet sublevels, are typically specific and intrinsic to every chromophore.

Since pulsed MASER operation has been demonstrated for pentacene: *p*-terphenyl, previously with 350 μs^1^ and 20 μs^2^ and here with 5 ns excitation pulses, clearly the intrinsic physical properties of this system are appropriate over a wide range of excitation duration and importantly at room temperature. However, when considering how these properties could be optimized the choices are limited. A straightforward chemical modification is to replace the protons on pentacene and *p*-terphenyl by deuterons. This is known to slow relaxation times in EPR and reduce linewidth. However, the hyperfine coupling is reduced to second order in zero-field, so this advantage found in high magnetic field is largely negated at zero-field^7^. Furthermore, deuteration of pentacene is challenging, and therefore, expensive so this approach is unlikely to be economic for practical applications.

Higher output power could also be achieved by increasing the concentration of the pentacene. In the experiments presented here, the concentration of pentacene in *p*-terphenyl is an order of magnitude below the estimated 0.1% mol/mol upper solubility limit for this system. This limits the number of molecules available for excitation. This is especially taxing when short excitation pulses are employed since each pentacene molecule has a single chance to become excited for each laser pulse. From this analysis it can be concluded that the maximum MASER power output could be substantially increased if the saturation limit of pentacene in *p*-therpenyl, imposed by crystal growth melt methods, could be overcome by alternative growth methods. A further problem is that at higher pentacene concentration and presumably using a more powerful laser, more heat will be dissipated in the sample which, as *p*-terphenyl is a poor conductor of heat (melting point 487 K, heat capacity proportional to temperature, C_p_ = 0.94 × T J K^−1^ mol^−1^)^13^, will warm up potentially leading to instability of the MASER output and, over the longer term, sample degradation. Indeed, the experiments reported here were performed with 2 Hz repetition rate in order to avoid these effects.

Other candidate aromatic molecules in other hosts may also have the suitable properties to be used as the gain medium for a maser, as reported in our recent density functional theory study^3^. Although the EPR literature includes several potential molecules, such as methylene blue, with the correct spin-polarization, much of the data have been presented in frozen solution at cryogenic temperatures, so alternative hosts, such as polymers or single crystals which are rigid at room temperature are also required^14^,^15^. The recent suggestions that nitrogen-vacancies centers in diamond^4^ and defects in silicon carbide^5^ would be potential gain materials for a maser appear promising, particularly as the thermal conductivity and durability of these materials are without doubt better than *p*-terphenyl.

The final components affecting performance and efficiency of an organic-based room temperature MASER are the microwave resonator and the pump source employed. Not only does the microwave resonator select which ZFS transition will be amplified, but also it controls the magnitude of the amplification due to the Purcell effect^16^, the enhancement of the stimulated emission of a molecule when matched in a resonant cavity, varies linearly with the resonator quality factor and inversely on its mode volume. The resonator *Q* factor also affects the time response of the MASER emission as shown by Equation 2. The technical details of the resonator used here have been described in detail previously (see ref. 2). However, in the light of the experiments reported here, potentially, other resonator designs that have different geometries and materials such as Fabry-Perot should be considered.

Previous publications have shown that pentacene-based MASERs can be operated also with flash-lamps and dye-lasers^1^,^2^. Although here we used an OPO-pumped Nd:YAG laser that allows tuning of the excitation wavelength to the optimum for the system, and produces intense nanosecond pulses allowing the spin dynamics to be examined in detail, for technological applications, the next step would be to move towards inexpensive and integrated diode lasers as pump sources.

## Materials and Methods

### Crystal growth

Pentacene commercial powder (TCI Europe nv) was vacuum purified and p-terphenyl commercially available powder (Alfa Aesar, 99 + %, AL4833) was zone-refined prior to use. A finely mixed powder, 0.006% mol/mol pentacene in *p*-terphenyl, was prepared and sealed in a 3 mm inner diameter surface modified quartz ampoule with vacuum level around 10^−3^ mbar. At one end of the ampoule, a sharp tip was made in order to promote self-seeding. The inner wall surface of the ampoule was decorated with *1H, 1H, 2H, 2H-perflouorodecyltrichlorosilane* (FDTS) and cleaned thoroughly using solvents (acetone, anhydrous isooctane, isopropanol and distilled water) in conjunction with an ultrasonic bath.

The pentacene: *p*-terphenyl crystal was grown by zone melting technique. To maintain and control the temperature of the laboratory-made furnace a Eurotherm 3216 temperature controller and a TE10A power controller were used. The melt-zone temperature was set at about 230 °C while the remaining region at 200 °C. The ampoule was dropped at a rate of around 1 mm per hour through the furnace using a gear motor. Thereafter, the furnace was cooled down at 1 °C/hour to room temperature and the ingot was retrieved. During the process, the pentacene dissolved in the molten zone, was swept upwards, creating a concentration gradient from low to high. The portion of the ingot with the most intense pink colour was selected for the experiments reported in the present paper. The *c*-plane of the monoclinic crystal structure was determined to be parallel to the crystal long axis.

### MASER device

The resonator used for this MASER has been described in detail in ref. 2. Briefly, it consists of three basic units: (i) a cylindrical copper resonator (diameter 42 mm, height 18 mm) equipped with a microwave port (a coaxial cable ended with a loop, coupling coefficient, *k* = 0.3, corresponding to −5.2 dB) to couple out microwave power generated by stimulated emission; (ii) a polished hollow cylinder of STO (strontium titanate, SrTiO_3_) single crystal (SurfaceNet GmbH) grown by the flame fusion method; and (iii) the pentacene: *p*-terphenyl crystal described above. The STO crystal (outer diameter 10.7 mm, inner diameter 3 mm, height 11 mm) supported a magnetic mode volume (V_m_) of 0.40 cm^3^. V_m_ is defined as the ratio of the stored magnetic energy within the cavity to the maximum magnetic density, as follow: V_m_ = *μ*_0_∫_V_|**H**(**r**)|^2^d*V/μ*_0_|**H**(**r**)|^2^. The pentacene: *p*-terphenyl crystal was cut and polished so to have the molecular *y*-axis of the pentacene molecules parallel to the cylindrical axis of the resonator, i.e. the direction of the magnetic field of the resonator mode. By means of an adjustable tuning plate, the resonator frequency could be matched to the X ↔ Z zero-field transition frequency at about 1.45 GHz. To estimate the maser output power coupling coefficient, a second very weakly coupled microwave port was introduced into the maser cavity. This, together with the use of a vector network analyzer (VNA, Hewlett Packard 8722ET 50 MHz–40 GHz), allowed a two-port transmission measurement to yield −27 dB as the coupling coefficient of the weakly coupled port. The same set-up was used to measure the loaded *Q*-factor of the maser resonator including pentacene: *p*-terphenyl crystal. The *Q*-factor is a dimensionless parameter that represents the ratio between the energy stored and the energy loss per cycle. In the present case it was found to correspond to 4900 and to be the same in the dark and under laser illumination.

### Optical excitation system

The light excitation was provided by a Surelite OPO pumped by the third harmonic of a Nd:YAG laser (5.5 ns pulse length and maximum repetition rate of 20 Hz). The OPO average emission power at each wavelength was measured with a pyroelectric high-power volume absorber detector (UP19K-15S-VR-D0). The OPO wavelength was determined by an Ocean Optics USB400 UV-Vis portable spectrometer. The laser energy was varied by adjusting the voltage of the flash lamp in the range from 1.06 to 1.01 kV. Before entering the MASER, the laser pulse passed through a beam splitter (95% transmission) so that the amplitude of a photodiode response could be used to measure the power profile of each pulse. Each set of measurements was conducted at a repetition rate of 2 Hz.

### MASER measurements

A schematic representation of the experimental set-up is given in Fig. 1c. In all measurements a Hewlett Packard 8722ET 50 MHz–40 GHz vector network analyzer (VNA) and a Keysight MSXO6004A oscilloscope (20 G samples s^−1^; 6 GHz bandwidth) were used. The frequency and power of the MASER output were extracted from the experimental signal using the Spectrogram function in Matlab™.

### MASER threshold measurement

The MASER resonator was tuned in transmission mode to the maximum of the X ↔ Z zero-field transition at 1.4493 GHz, by means of the VNA. The resonator was under-coupled with the coupling parameter, *k* = 0.3 (−5.2 dB). The *k* value used provided a good balance between losses and amplitude of the observed MASER signal. Subsequently, the resonator was disconnected from the VNA and the MASER output coming from the coupling port was directly connected to the oscilloscope, which was externally triggered by the Q-switch output of the laser. One hundred consecutive single-shot measurements were collected to account for the variability in the OPO emission and to allow statistical analysis to be performed. In order to determine the MASER threshold, the same measurement was repeated for different pumping power energies, from 2.4 to 8.8 mJ/pulse. The experimental data, consisting of MASER and photodiode output, were retrieved from the oscilloscope and collected using a lab-written Python program. All measurements were conducted at room temperature.

### MASER seeding measurement

Similar to threshold measurements, the MASER resonator was tuned to the maximum of the X ↔ Z zero-field transition and the MASER output was fed into the oscilloscope directly. This time, the VNA was not disconnected and a continuous microwave radiation, at 1.45 GHz, was injected into the MASER resonator through the very weakly coupled port (coupling coefficient: −27 dB). The same measurement was repeated for different nominal seeding powers, from −70 to −20 dBm. One hundred consecutive single-shot measurements were collected to account for the variability in the OPO emission and to allow statistical analysis to be performed. The optical pumping energy was kept constant at 6.7 mJ/pulse and all measurements were conducted at room temperature.

### Statistical analysis

To understand the nature of the observed distributions of MASER parameters observed in each set of measurements, histograms of the laser energy, peak power, delay and frequency at each mean laser energy were constructed and compared to Gaussian distributions (one-sample Kolmogorov-Smirnov test) using Matlab™ built-in statistical app.

### Derivation of stimulated Einstein coefficient for linearly polarized microwave field

Considering transitions between the X and Z states of the triplet, at zero-field the triplets can be written in terms of the high field basis, 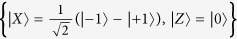. The microwave field within the cavity interacts with the S = 1 spins, resulting in a perturbation to the Hamiltonian of 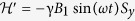 where *γ* is the gyromagnetic ratio, *B*_1_ is the microwave magnetic field, *ω* is its angular frequency and *S*_*y*_ is the spin operator which can be decomposed into ladder operators *S*_+_ = *S*_*x*_ + *iS*_*y*_ and S_−_ = *S*_*x*_ − *iS*_*y*_. Using time-dependent perturbation theory (Fermi’s Golden Rule) and the rotating wave approximation (RWA) allows the transition probability per unit time to written:


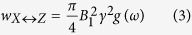


where *g*(*ω*) is the normalised shape function for the *X* ↔ *Z* transition, which at the spin frequency *ω*_*s*_ is *g*(*ω*_*s*_) = *T*_2_/*π*, where *T*_2_ is the homogeneous spin-spin decoherence time. The microwave magnetic field energy density can be expressed in terms of the cavity mode energy (*nħω*) and mode volume:


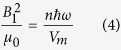


where *n* is the cavity mode photon number and *V*_*m*_ is the magnetic mode volume:


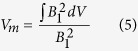


For broadband excitation, when the exciting field is broadband compared to the transition linewidth, i.e. the electromagnetic field energy density does not vary over the spin transition linewidth, this leads to a transition rate proportional to *n*, from which the Einstein coefficient *B*^*n*^ can be found:


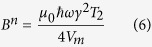


The mode volume was calculated to be 0.25 cm^3^ and the spin-spin decoherence time *T*_*2*_ ~ 2.9 μs was taken from^17^. This yields an estimate of the *B*^*n*^ coefficient of 11.3 × 10^−8^ s^−1^ assuming pentacene to be perfectly aligned to the resonator mode. If the two non-equivalent sites are taken into account and only one site is aligned to the resonator mode, the predicted value reduces to 8.5 × 10^−8^ s^−1^.

## Additional Information

**How to cite this article**: Salvadori, E. *et al*. Nanosecond time-resolved characterization of a pentacene-based room-temperature MASER. *Sci. Rep.* **7**, 41836; doi: 10.1038/srep41836 (2017).

**Publisher's note:** Springer Nature remains neutral with regard to jurisdictional claims in published maps and institutional affiliations.

## Supplementary Material

Supplementary Video 1

Supplementary Information
