# Supplementary material for: Nanosecond time-resolved characterization of a pentacene-based room-temperature MASER
Source: Sci Rep. 2017 Feb 7;7:41836. doi: 10.1038/srep41836 (PMC5294447; doi:10.1038/srep41836)
Supplement: Supplementary Information [file srep41836-s2.pdf]

Supplementary Information

Nanosecond time-resolved characterization of a pentacene-based room-temperature MASER

Enrico Salvadori[1,2,3,*], Jonathan D. Breeze[4], Ke-Jie Tan[4], Juna Sathian[4], Benjamin Richards[4], Mei Wai Fung[2], Gary Wolfowicz [1], Mark Oxborrow[4], Neil McN. Alford[4] and Christopher W. M. Kay[1,2,*]

[1] London Centre for Nanotechnology, University College London, 17-19 Gordon Street, London WC1H 0AH, UK

[2] Institute of Structural and Molecular Biology, University College London, Gower Street, London WC1E 6BT, UK

[3] School of Biological and Chemical Sciences, Queen Mary University of London, Mile End Road, London E1 4NS, UK

[4] Department of Materials, Imperial College London, London

Supplementary Video 1. Real time single-shot MASER emission

Top panel: One hundred consecutive MASER emission time traces recorded at 3.3 mJ/pulse average laser energy and 2 Hz repetition rate, direct detection without signal averaging. The inset shows an enlargement of the signal and fitting to a sine function.
Middle panel: Frequency-power analysis of each MASER emission time trace (Matlab™ *spectrogram* function).
Bottom panel: Power output of each MASER emission time trace taken at the maximum of the frequency-power spectrum.

Supplementary Figure 1. Statistical analysis

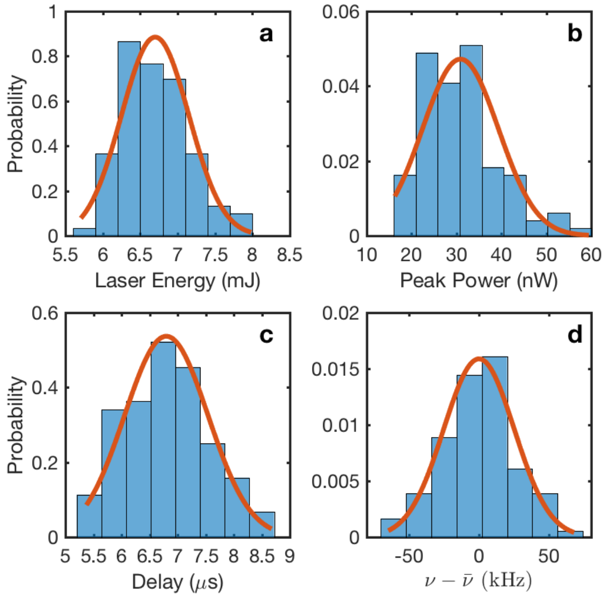

Histograms of the distributions of (a) laser energy, (b) peak power, (c) delay and (d) frequency of MASER emission at 6.7 mJ/pulse pump energy. The red lines represent Gaussian fits. One-sample Kolmogorov-Smirnov tests confirm that none of the distributions deviate significantly from normality.
